# Visualizing Sound Emission of Elephant Vocalizations: Evidence for Two Rumble Production Types

**DOI:** 10.1371/journal.pone.0048907

**Published:** 2012-11-14

**Authors:** Angela S. Stoeger, Gunnar Heilmann, Matthias Zeppelzauer, André Ganswindt, Sean Hensman, Benjamin D. Charlton

**Affiliations:** 1 Department of Cognitive Biology, University of Vienna, Vienna, Austria; 2 Gfai tech GmbH, Berlin, Germany; 3 Institute for Software Technology and Interactive Systems, Vienna University of Technology, Vienna, Austria; 4 Department of Zoology and Entomology, Mammal Research Institute, University of Pretoria, Pretoria, South Africa; 5 Section of Reproduction, Department of Production Animal Studies, University of Pretoria, Onderstepoort, South Africa; 6 Adventures with Elephants, Bela Bela, South Africa; 7 School of Psychology, University of Sussex, Brighton, United Kingdom; University of Milan, Italy

## Abstract

Recent comparative data reveal that formant frequencies are cues to body size in animals, due to a close relationship between formant frequency spacing, vocal tract length and overall body size. Accordingly, intriguing morphological adaptations to elongate the vocal tract in order to lower formants occur in several species, with the size exaggeration hypothesis being proposed to justify most of these observations. While the elephant trunk is strongly implicated to account for the low formants of elephant rumbles, it is unknown whether elephants emit these vocalizations exclusively through the trunk, or whether the mouth is also involved in rumble production. In this study we used a sound visualization method (an acoustic camera) to record rumbles of five captive African elephants during spatial separation and subsequent bonding situations. Our results showed that the female elephants in our analysis produced two distinct types of rumble vocalizations based on vocal path differences: a nasally- and an orally-emitted rumble. Interestingly, nasal rumbles predominated during contact calling, whereas oral rumbles were mainly produced in bonding situations. In addition, nasal and oral rumbles varied considerably in their acoustic structure. In particular, the values of the first two formants reflected the estimated lengths of the vocal paths, corresponding to a vocal tract length of around 2 meters for nasal, and around 0.7 meters for oral rumbles. These results suggest that African elephants may be switching vocal paths to actively vary vocal tract length (with considerable variation in formants) according to context, and call for further research investigating the function of formant modulation in elephant vocalizations. Furthermore, by confirming the use of the elephant trunk in long distance rumble production, our findings provide an explanation for the extremely low formants in these calls, and may also indicate that formant lowering functions to increase call propagation distances in this species'.

## Introduction

Individual and species-specific mechanisms of sound production determine the vocal characteristics accessible to receivers, and therefore, to natural and sexual selection. This evolutionary interconnection of voice production, acoustic output and function makes it necessary to understand basic sound production mechanisms when studying animal communication [1]. Mammalian vocal production at the level of the larynx is thought to follow the principles of the myoelastic-aerodynamic theory of human phonation [2]. Sound waves generated by vocal fold vibration in the larynx pass through the vocal tract, which contains air in the pharyngeal, oral, and nasal cavities, amplifying certain frequencies termed formant frequencies (or formants), before radiating into the environment. Formant frequency values are determined by the length and shape of the vocal tract, with longer vocal tracts producing lower, more closely spaced formants. Furthermore, formants are reliable cues to body size in several mammals [3]–[9] due to a close relationship between the frequency spacing of the formants, the caller's vocal tract length and overall body size. This, together with demonstrations of formant perception by nonhuman mammals in general [10]–[14] and interspecific perception [15], [16] in particular, suggests that formants may provide a universal cue to body size in vertebrates [17].

Intriguing morphological adaptations to elongate the vocal tract in order to lower formants occur in several species, with the size exaggeration hypothesis [18] being proposed to justify most of these observations (e.g. birds [19]; red deer, *Cervus elaphus*, [20]; big cats, *Panthera sp.* [21]; Goitred gazelles, *Gazella subgutturosa* [22]; koalas, *Phascolarctos cinereus* [4]; elephant seals, *Mirounga leonina* [8]). An alternative explanation, however, is that lowering formants aids long-distance call propagation [23]. Indeed, whereas formant variation in African elephant (*Loxodonta africana*) rumbles appears to be functionally relevant in this species' vocal communication system [24], [25], the adaptive significance of the extremely low formant frequencies of African elephant rumbles [23] is unknown i.e. it is not known whether the very low formants of elephant rumbles reflect sexual selection pressures to sound larger, or natural selection pressures to maximize call propagation distances. Furthermore, while the very low formants of elephant rumbles strongly implicate that the elephant trunk is involved in sound production [23], [26] (the un-extended trunk length of an adult female African elephant is around 1.7 to 1.8 m [27]) it is not known whether elephants emit these vocalizations exclusively through the trunk, or whether the mouth is also involved in rumble production [23], [26], [28]–[30].

Elephant rumbles are frequency-modulated, harmonically rich vocalizations that are known to convey information about age, individuality and arousal state [23], [29], [31]–[33]. Female African elephant rumbles are also thought to be used for group cohesion and coordination [31]) and have been described as having a graded within-call type variation; however, no strong evidence for rumble subtypes based on structural variation has been documented [29]. Even less is known about male African elephant rumbles: the so-called “musth-rumble” is constantly produced by male elephants in musth (a condition in bull elephants characterized by increased aggressive behaviour and elevated androgen levels) and is suggested to acoustically advertise the animal's hormonal state [33]. Indeed, whereas the potential adaptive functions of African elephant rumbles have received a lot of attention, to date, the physiological mechanisms of vocal production have been largely neglected (but see: [30]).

In this study we used a novel sound visualization technique (an acoustic camera) to record five captive African elephants during spatial separation and subsequent reunions (bonding) in order to investigate whether rumbles are produced using the trunk and/or the mouth in these specific contexts. The acoustic data was then used to compare the spectral structure of rumbles given in the two contexts, and to determine whether it is possible to automatically classify these rumble variants using a smoothed spectral representation based on Linear Predictive Coding (LPC) for both rumble variants and machine learning. Our findings will improve our knowledge of African elephant rumble production, and may help to confirm the role of the elephant's trunk in producing the extremely low formants observed in these calls.

## Methods

### Data collection

#### Study subjects and housing

The subjects in this study were five African elephants (three females and two males) aged between 9 and 17 years (Table 1) located at Adventures with Elephants, Bela Bela, South Africa. These elephants had been captured during culling operations between 4 and 5 years ago. The elephants were fully habituated to human presence and free to roam around in an area of 300 ha.

**Table 1:** Results of the acoustic analysis.

	Chichuru	Chova	Messina	Nuanedi	Shan
Sex	male	male	female	female	female
Age in years	15	17	9	10	13
**N rumbles nasal (% separation context)**	**26 (92%)**	**13 (77%)**	**25 (96%)**	**29 (93%)**	**22 (100%)**
Mean duration ± SD	1.9±1.3	1.4±0.6	2.8±1.5	3.1±1.8	2.8±0.8
Mean F0 ± SD	16.5±1.9	16.7±0.6	19.5±3.2	19.57±2.3	20.5±3.2
Mean F1 ± SD	40.1±4.9	39.5±9.5	45.3±30.7	42.6±7.4	42.0±23.2
Mean F2 ± SD	117.9±11.2	121.6±7.4	140.5±75.8	129±10.1	139.1±89.6
Mean SPL ± SD	52.0±4.7	43.7±8.1	51.1±7.7	53.5±4.8	48.7±6.5
VTL (m)	*2.24*	*2.13*	*1.84*	*2.03*	*1.80*
**N rumbles oral (%bonding context)**	**0**	**0**	**21 (86%)**	**21 (72%)**	**10 (100%)**
Mean duration ± SD			1.66±0.8	2.0±1.3	1.2±0.7
Mean F0 ± SD			24.7±4.4	30.2±2.4	22.7±4.8
Mean F1 ± SD			162.1±44.6	162.0±29.8	176.1±16.4
Mean F2 ± SD			381.1±90.1	397.89±20.3	453.1±35.0
SPL ± SD			74.5±10.1	75.6±2.9	69.2±8.4
VTL (m)			*0.79*	*0.74*	*0.63*

The age and the sex of each recorded individual, the number of orally and nasally emitted rumbles (and the percentage of those recorded in each context, respectively), and the mean duration, mean fundamental frequency, mean formant frequency values 1 and 2, and mean sound pressure level (SPL) ± SD of rumbles per individual are presented. The estimated vocal tract length (VTL) for each individual based on the spacing in Hz between formants 1 and 2 for nasal and oral rumbles is also given.

#### Acoustic camera recordings

Recordings were captured over 4 days (22 November to 25 November 2011), with a total of 20 h of data collected during this period. The temperature during the recording sessions varied between 20 and 25°C, and recordings were only captured when wind speed was low. Two recording session were conducted at around 8 a.m and 4 p.m each day.

To visualize sound emission we used an acoustic camera star 48 array [34]. The star-shaped array has a span width of 3.4 m with 48 microphone channels (Sennheiser Electric-Capsules with MicBus microphone connectors: dynamic range 35…130 dB and 10 Hz … 20 kHz; the microphone capsules are used in connection with a symmetrical output buffer. The buffer contains first order (−6 dB/Octave) filters for bandwidth limitation. The low cut is set to 100 Hz@-3 dB and the high cut is set to 100 kHz@-3 dB). A video camera (Baumer TXG06C) was integrated into the array so that video and acoustic data could be captured at the same time. Additional trigger signals from the video camera allowed us to synchronize video images and acoustic data (the camera delivered the actual exposure times during recording of the video images as trigger pulses).

**Figure 1:**
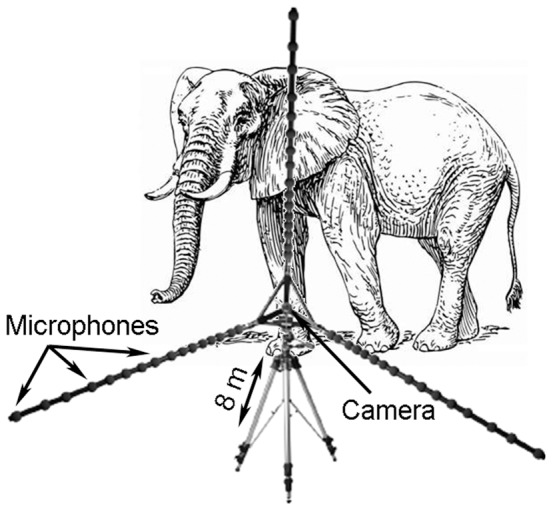
Experimental setup. The microphone array with 48 channels was connected to the recorder and a Laptop, and placed around 8 meters from the focal elephant.

The acoustic and video data were recorded using a mcdRec data recorder [34] at a sampling rate of 48 kHz. During recordings, the microphone array was positioned approximately 8 m (using a laser rangefinder) from the elephants (for the experimental setup, see Figure 1). Due to the data volume created by the acoustic camera, single recording sessions with this system varied between 30 and 180 s. A pre-recording trigger was set (depending on the lengths of the recordings) so that the record button could be started once the elephant(s) had started to vocalize. Thus, when the record button was pressed, everything that took place in the previous 30 s was also recorded and saved.

#### Recording contexts

Vocalizations were recorded in two distinct social contexts: spatial separation and subsequent reunions (bonding). The experimental sessions were carried out alongside the daily training routines (which typically involved chaining the elephants on one leg, a health check, and sometimes the training of particular commands). During the recording sessions elephants were chained, provided with pellets, and the keepers did not interact with them. Recordings started with the separation context. In this context the focal elephant was chained by one leg whilst the remaining elephants were walked out of sight (by the keepers) to the savannah, 500–700 m away. The focal elephant was then recorded for 5 minutes (separation context). For the bonding contexts, the other elephants were reunited with the focal elephant one by one, with the order of individuals brought back to the focal elephant alternated. Initially, keepers accompanied the incoming elephant until they had visual contact with the focal animal before allowing the incoming elephant to approach the focal animal alone. This resulted in a bonding ceremony, which usually involved the incoming elephant running towards the focal elephant and vocalising, raising the tail, spreading the ears and producing temporal gland secretions. Once reunited, the elephants remained close to each other. During this period they would entwine trunks, slightly push or back towards each other, and sometimes urinate and/or defecate [35]). Each elephant served as a subject in the experiment once a day. However, if a reunited elephant vocalized in front of the acoustic camera (within the approximate range of 8 m), those vocalizations were also captured.

### Data analysis

#### Acoustic video analysis

The acoustic videos were analyzed using the software Noise Image [34]. The initial data, which were originally saved as channel files (*.chl), were reconverted into 2D acoustic movie files (amo-format, 25 f/s). This technology analyses the actual sound scene, which consists of a superposition of different sound sources, into a visual sound map. The basic principle relies on accurately calculating the specific runtime delays of acoustic sound emissions radiating from several sources to the individual microphones of the array [36]. An acoustic map of the local sound pressure distribution at a given distance is calculated using the acoustic data of all simultaneously recorded microphone channels. The sound pressure level (SPL) is displayed by colour coding. The automatic overlay of optical image and acoustic map allows the locations of dominating sound sources to be identified.

The time function ƒ of a point x = (x′, y′, z′)^T^ on the image plane was reconstructed by delay-and-sum beamforming [37] according to equation 1. Here, *t* denotes time, *M* is the number of microphones in the array, the *w*_i_ are (optional) shading weights, the *f_i_* are the recorded time functions of the individual microphones, and the Δ_i_ are the appropriate relative time delays, calculated from the absolute run times π_i_ as Δ_i_ = π_i_−min (π_i_). The absolute run times are determined by π_i_ = |**r**_i_|/v, where v is the speed of sound in air and |**r**_i_| is the geometrical distance from microphone number i to the point of interest **x**.
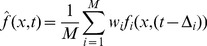
(1)


The effective sound pressure at point **x** (L_p_ dBSPL) is determined using equation 2; every individual pixel is then coloured corresponding to its effective value and a given colour table. In equation 2, *n* is the total number of discrete time samples taken into account in estimating the effective value, ƒ is the reconstructed time function of equation 1 of the sound pressure at location **x**, and t_k_ is the time value at a discrete sample index *k*.
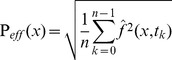
(2)


The acoustic movie files were visually analyzed and the vocalizations were investigated frame by frame. The location of sound emission (nasal or oral) was visually identified for each recorded vocalization by the first author and a second observer (reaching 98% agreement). Due to the distance between the trunk tip and the mouth, it was possible to clearly distinguish between oral and nasal sound emission. The rumble was allocated as being nasally emitted when the most intensive colouring was located around the trunk tip, and orally emitted when the most intensive colouring was located around the mouth. We analyzed 179 rumble vocalizations. Peak SPL during the vocalization was quantified using the maximum value at the middle of the vocalization. Selected frames were exported from the acoustic movie to JPG-Format. For presentation, parts of the acoustic movies were exported to AVI-Format in slow motion (without sound, 5 f/s) and real time in 2D (see Movies S1, S2, S3, S4).

#### Acoustical analysis

For acoustic analysis, we exported the acoustic signal (in stereo) of each rumble video (in which we could clearly identify whether sound emission was nasal or oral) to WAV format. Acoustic analyses were performed using Praat 5.0.29 DSP package [38]. The fundamental frequency was measured over the entire utterance with the “to pitch (ac)” command (time step 0.01, window lengths 0.4 s). The settings for pitch extraction were calibrated by inspecting the accuracy of the pitch line generated by Praat on spectrograms (minimum frequency 10 Hz; maximum frequency 35 Hz in nasal and 40 Hz in oral rumbles). The minimum, the maximum, the range (max-min) and the mean ± SD fundamental frequency were extracted. In addition, minimum, maximum and mean ± SD duration of rumbles were measured from the waveform.

Because formants 3 and 4 could not be consistently measured we only considered the lower two formants in the analysis. To examine formants 1 and 2 we segmented 0.5 s of each rumble (starting from the mid point of the vocalization). The rumble segments were then re-sampled to 6000 Hz and LPC was performed on the spectra of the annotated time units. Using a linear tube model closed at one end (partially closed at the vocal folds) and open at the other end (mouth or trunk), the formant locations (F) are given by equation 3 (Table 2), where *n* is the formant number, *c* is the speed of sound (350 m/s), and vocal tract length (VTL) in meters, using an estimated VTL of 0.75 m for the oral rumbles and 2.5 m for the nasal rumbles [26]. These estimates are derived from data on a large sample of mandibles from female African elephants (ranging in lengths from about 45 cm at age 15 to 60 cm at age 60) made by Laws and colleagues [39], taking into account that the larynx is positioned posterior to the mandible and that the lips protrude past the anterior process of the mandible, as well as considering the trunk lengths of about 1.7 meter [27]. Based on the predicted formant locations derived from equation 3, the number of peaks was set to ‘2 in 400 Hz’ for oral rumbles, and ‘2 in 150 Hz’ for nasal rumbles (Table 2). The VTL of each individual for nasal and oral rumbles (only nasal rumbles for males) was estimated using equation 4 [6], where *c* is the speed of sound (350 m/s) and *ΔF* the formant spacing.

**Table 2:** Predicted formant values of oral and nasal rumbles in African elephants (after [26]).

Equation 3	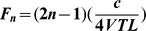	
Formant	Predicted formant value (Hz) for nasal rumbles	Predicted formant value (Hz) for oral rumbles
Formant 1	35.0	116.7
Formant 2	105.0	350.0

The equation to calculate formant values based on VTL and the predicted values of formants 1 and 2 for oral and nasal rumbles in African elephants.


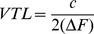
(4)

#### Statistical analysis

Linear mixed-effect models (LMMs) [40] were used to investigate acoustic variation across nasal and oral rumbles in the three females Shan, Nuanedi and Messina. Separate LMMs were run in which the dependent variables were the first formant, the second formant, call duration, mean fundamental frequency and the sound pressure level. For each model, location of sound emission was entered as a fixed factor (oral versus nasal), individual identity as a random factor, and age as covariate. A scaled-identity covariance structure was used for all the LMMs, and we used a model selection criteria based on the Akaike's Information Criteria (AIC), in which the model having the lowest AIC value is chosen (*sensu* [41]). Age had no significant effect on the results, and the lowest AIC values were achieved when entering only location of sound emission as the fixed factor and individual identity as the random factor (omitting age). To ensure that the test compared likelihoods based on the same data, the maximum likelihood estimation method was used to test the hypotheses [42]. All statistical tests above were performed in PASW Statistics 18.0.

#### Automatic classification

For the automatic classification, we first computed a numerical representation for each nasal and oral rumble, applying a sliding window to each sound sample with a window size of 300 ms and a step size of 30 ms. For each window we computed the LPC-smoothed spectrum in the range of 0 Hz to 500 Hz (model order 8). The result was a two-dimensional (2D) LPC spectrogram that represents the smoothed spectral shape over time preserving the formant structure of the call. Note that we applied the *same* parameters for both types of rumbles.

Classification techniques such as Linear Discriminant Analysis (LDA) require that each sound sample is represented by a single vector. We computed the average LPC spectrum over time to obtain one representative (and time invariant) numerical vector for each sound sample. We then sub-sampled the vector to 26 components to obtain a more robust and compact representation for classification. We first employed LDA for classification. In order to evaluate the dependency of classification performance on a particular classification technique we further applied a linear Support Vector Machine (SVM) [43], and Nearest Neighbour Classification (NN).

For the evaluation of automatic classification performance, we first split the data set into a training set (1/3 of all samples) and an evaluation set (2/3 of all samples). We applied k-fold cross-validation (k = 10) on the training set to evaluate stable parameters for three different classifiers and to reduce the dependency of the classifiers from the training data. All experiments were performed in MATLAB R2012a.

## Results

### Sound visualization experiments

**Figure 2:**
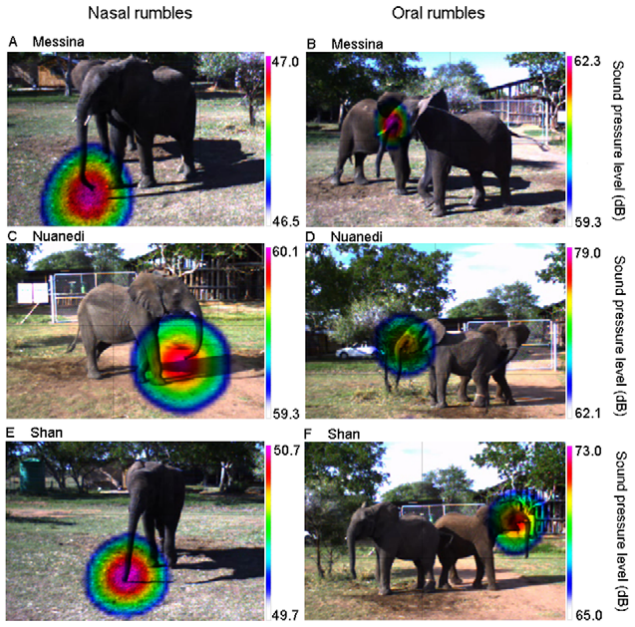
Sound visualization of African elephant rumbling vocalizations. Examples of nasal and oral rumbling vocalizations from three female elephants, Messina, Nuanedi and Shan. Figures A, C and E give examples of nasal rumbles, B, D and F give examples of oral rumbles.

Using the acoustic camera, we captured 179 rumble vocalizations from 5 African elephants (three females and two males). Detection of sound emission was very accurate and could be clearly allocated in 167 rumbles. The 12 cases (∼7%) in which the location of sound emission could not be clearly allocated were either due to high levels of background noise resulting in a diffuse acoustic video (10 times), or because the trunk moved towards the mouth and the location of sound emission could not be reliably discriminated (two times). Of the 167 rumbles in the analysis, 115 were uttered nasally (sound emission through the trunk) and 52 were emitted orally (from the mouth). Furthermore, 92% of the rumbles were emitted nasally during the separation context and 84% of the rumbles were emitted orally during bonding situations. Orally emitted rumbles were only produced by females (Figure 2) and males mainly vocalized during the separation context, with only five nasal rumbles recorded in the bonding context.

### Acoustic analysis

**Figure 3:**
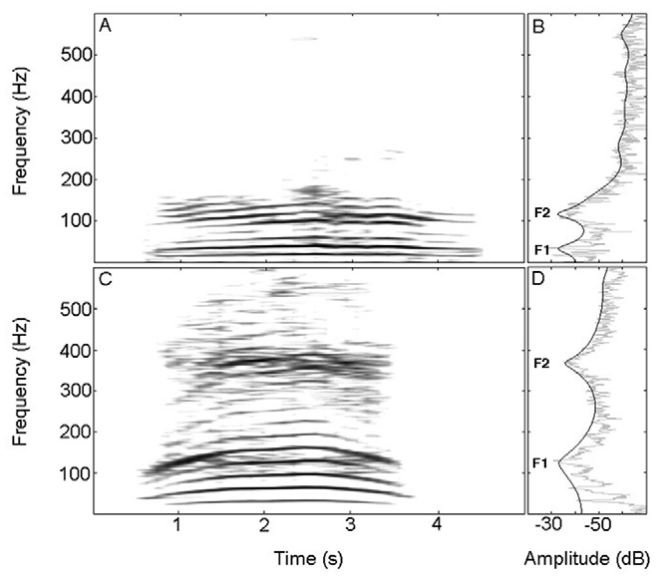
Spectral characteristics of nasal and oral rumbles. Spectrograms and power spectra showing an example of a nasal (A, B) and an oral (C, D) rumble, indicating formant positions (both rumbles uttered by Nuanedi, 10-year-old female).

The values of formant 1 and formant 2 for the nasal rumbles (formant 1±SD = 39.79±5.78 Hz and mean formant 2±SD = 128.76.79±32.57 Hz) and oral rumbles (mean formant 1±SD = 169.21±25.61 Hz and mean formant 2±SD = 415.20±47.71 Hz) of the three female African elephants differed significantly (see Figure 3; LMM: formant 1: F_1,166_ = 849.006, p<0.001; formant 2: F_1,166_ = 730.004, p<0.001). These results accord well with the values predicted by a simple tube model closed at one end (closed at vocal folds) and open at the other end (mouth or trunk, Table 1), indicating that the observed spectral peak frequencies are very likely to be formants (vocal tract resonances). In addition, the duration of nasal rumbles was significantly greater than oral rumbles (mean ± SD nasal rumbles = 2.94 s±1.6; mean ± SD oral rumbles: 1.79 s±1.1; LMM: F_1,166_ = 15.786, p<0.001). The mean fundamental frequency was significantly lower in nasal rumbles (mean ± SD = 19.7±2.7 Hz) than in oral rumbles (mean ± SD = 26.9±4.6 Hz; LMM: F_1,166_ = 98.373, p<0.001). Finally, the sound pressure level (L_p_ dBSPL @ 8 m) was significantly lower in nasal rumbles (mean SPL ± SD = 51.9±6.22 dB) than it was in oral rumbles (mean SPL ± SD = 74.45±7.49 dB; LMM: F_1,166_ = 229.296, p<0.001).

### Automatic classification

**Figure 4:**
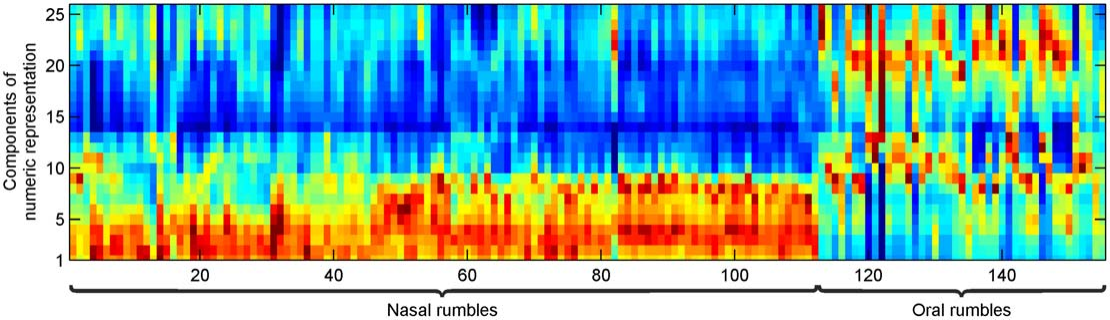
Automatic classification of rumbling vocalizations. Numerical descriptors (averaged LPC spectrum) for all sound samples in the experiments. Each column of the matrix represents one descriptor of a rumble. Red represents spectral peaks while blue represents low spectral components. The descriptors of the nasal and oral rumbles show significantly different characteristics.

The automatic classification was performed using an LPC-based spectral representation for both types of rumbles. Figure 4 gives the representational vectors for each nasal and oral rumble in our dataset, clearly showing the different spectral characteristics of both types of rumbles. All classifiers generalized well to the underlying data (training set) with standard parameters. Next, we applied the trained classifiers on the evaluation set (which has not been used during training) and computed the accuracy of classification. We obtained a classification accuracy of 99% with LDA, meaning that only 1 vocalization was misclassified. In order to investigate the dependency of this result on the employed classification technique, we evaluated the classification accuracy of two further classifiers (Support Vector Machine and Nearest Neighbour Classifier). Both classifiers yielded accuracies above 97% similarly to LDA, demonstrating that the high classification accuracy was not dependent on a particular classification technique. Similar results were obtained when we exchanged training and evaluation sets revealing that the dependency of classification performance on the training data is low. This evaluation demonstrates that the oral and nasal rumbles could be distinguished with high accuracy by an automatic classification system without taking call specific characteristics (e.g. predefined formant frequencies) into account.

## Discussion

Using an acoustic camera array to visualize sound emission, we have demonstrated two types of rumbles in three sub-adult female African elephants: a nasally and an orally emitted rumble. In addition, nasal and oral rumbles in our data set varied considerably in their acoustic structure. In particular, the mean frequency spacing of the first two formants predicted the estimated lengths of the two vocal paths. This corresponded to a vocal tract length of about 2 m for nasal rumbles and about 0.7 m for oral rumbles in the investigated elephants (note that all were below the age of 17 years and not yet fully-grown). Thus, by using the nasal path, an elephant lowers it's formants by around threefold. However, because the elephants in our study were all sub-adults, we must exercise extreme caution when generalising our results to all age classes. Indeed, young elephants may simply tend to produce oral rumbles more often than adults. Nevertheless, preliminary results generated from a large sample of African elephant rumbles (Stoeger et al, unpublished data) indicate that adult female elephants do produce oral rumbles (although only verified by formant structure; see Figure S1 and S2) and hence, suggest that elephants (at least females) of all age classes might produce oral rumbles in certain situations.

In addition, we have also shown that the African elephants in our study produced the two different rumble types in two distinct contexts. Nasal rumbles predominated during contact calling, whereas oral rumbles were mainly observed during the social bonding context. In human speech, formants (particularly formants 1 and 2) provide the acoustic basis for discriminating vowels and thus, are a very important means of transferring information [44], [45]. The active modulation of the lower two formants also appears to play a role in referential calling in nonhuman primates such as hamadryas baboons (*Papio hamadryas*; [46]), gelada baboons (*Theropithecus gelada*; [47]), vervet monkeys (*Ceropithecus aethiops*; [48]), and Diana monkeys (*Cercopithecus diana*; [49]). Previous elephant studies have also documented formant variation with context and/or arousal: specifically, an upward shift in the second formant seems to alert other elephants to potential danger [24], and female elephants engaged in dominance interactions produce rumbles with lower formant dispersion (spacing) compared to rumbles produced in low affect contexts [25]. However, whether this formant variation is produced by switching from nasal to oral sound production, or whether a specific formant shifting can also be achieved by modulating structures of the nasal or oral vocal tract respectively, remains to be investigated.

Interestingly, the two bulls in our dataset only produced nasal rumbles (and mainly vocalized during the separation context), which might reflect the already reported sexual dimorphism in the vocal behaviour of African elephants (bulls are less vocal and less focused on social cohesion compared to females [31]). Although this result must be treated with caution due to the small sample size and the young age of these males, if bulls do produce nasal rumbles more often than oral rumbles, they may be maximizing the impression of their size with these vocalizations. Indeed, body size and age are important correlates of reproductive success in African elephant bulls [50], and male-male competition is likely to be an important selective force acting on the acoustic structure of male rumbles. Future research, therefore, should investigate whether formants in male rumbles are predictive of the caller's body size, and document the behavioural responses of male African elephants to playbacks of rumbles with different (and maybe resynthesized) formant values. It is noteworthy that the three female African elephants mainly produced nasal rumbles in the contexts of long distance contact calling. Accordingly, because lower frequencies typically propagate over greater distances [51] another interpretation for our findings might be that lowering formants increases call propagation distances in this species'.

The oral rumbles produced by the three females recorded during bonding situations also showed an increase in fundamental frequency compared to the nasal rumbles. Increased fundamental frequency is correlated with increased arousal state in many mammalian species [52], [53] including African elephants [25], [54] and the females often showed temporal gland secretion and displayed increased locomotion during bonding, both of which indicate higher arousal levels than during contact calling. In addition, female oral rumbles were considerably louder than those emitted through the trunk. Since nasal passages in most mammals are convoluted and filled with spongy absorptive tissue, nasal sounds are typically much quieter than oral sounds [55]. Indeed, cineradiographic data indicate that loud sounds are generally produced orally in all mammals studied so far (e.g., dog barks, goat bleats, pig squeals, or monkey chatters), with some softer sounds (e.g., dog whines or pig grunts) being produced nasally [55]. These observations argue against our contention that nasal rumbles are used for long distance communication though, because vocalizations with lower amplitude will obviously not propagate as far as louder calls. Nevertheless, it is worth noting that the nasal rumbles recorded during separation contexts in our experiments were directed towards con-specifics a maximum of 600–700 meters away, and that these calls might be expected to have a higher sound intensity when directed towards elephants over greater distances. Moreover, there may be an evolutionary trade off between lower frequencies and call amplitude, if the former results in better sound transmission of relevant frequencies. In addition, it is possible that lowering formants in rumbles makes the call perceptually louder to conspecific receivers, if African elephants are particularly sensitive to very low frequencies (as may be expected given the extremely low frequencies of elephant rumbles and the hearing sensitivity observed in an Asian elephant, *Elephas maximus* [56]). Playback experiments designed to test formant perception and the frequency range of best sensitivity in African elephants are now required.

To conclude, our results show that African elephants are able to vary their vocal path and dramatically lower formants in their rumble vocalizations, and that they might do this systematically according to context or motivation. It is important to note that formants are expected to vary due to the age/size of an elephant, individual morphological variations of the vocal tract, and probably due to context, motivation, arousal state and potentially, social rank. Furthermore, it may not be excluded that elephants switch from nasal to oral sound production (or the other way around) within a vocalization. Nevertheless, by showing that rumbles can be emitted via the trunk or mouth, the findings of the current study have furthered our knowledge of elephant vocal production, and how this impacts on the acoustic characteristics of elephant vocalizations. While our small sample size and the relatively young age of the study animals means we must exercise a degree of caution when generalizing these results, our findings should stimulate new research on this species vocal communication system. In particular, we suggest that future studies determine whether the formants present in African elephant rumbles consistently vary according to the size of the vocalizing animal, and also investigate the behavioural responses of male and female conspecifics to formant variation in rumbles. Re-recording experiments could also reveal whether any size-related formant information persists over relevant distances. Finally, by introducing a sound visualization method that has not previously been used in the field of bioacoustics, we have provided a methodological advance that could be used not only to identify callers in a wide range of species (e.g. when animals call in large colonies) but also to potentially investigate whether animals use their nasal or oral vocal tract in call production, as well as confirming whether calls are produced on expiration or inhalation. Future studies incorporating this novel technique are certainly warranted.

## Supporting Information

Movie S1 **Nasal rumble-25 fps-sound: Sound visualization of a nasal rumble. This movie shows the sound emission during a nasal rumble.**
(AVI)

Movie S2 **Nasal rumble-5 fps-slow-mo: Sound visualization of a nasal rumble in slow motion.** This movie shows the sound emission during a nasal rumble in slow motion (5 frames per second).
(AVI)

Movie S3 **Oral rumble-25 fps-sound: Sound visualization of an oral rumble.** This movie shows the sound emission during an oral rumble.
(AVI)

Movie S4 **Oral rumble-5 fps-slow-mo: Sound visualization of an oral rumble in slow motion.** This movie shows the sound emission during an oral rumble in slow motion (5 frames per second).
(AVI)

Figure S1 **Spectrograms and power spectra presenting two examples of rumbling vocalizations from a 29 year old female African elephant (Drumbo) recorded at the Vienna Zoo in 2003.** Recordings were captured with a condenser microphone AKG 480 B CK 62 and a DA-P1 DAT recorder. Figures A and B show a rumble recorded during spatial separation from a part of the group, and display the formant structure of a typical nasal rumble. Figures C and D show a rumble recorded during a bonding situation when the group was reunited, and resemble an orally emitted rumble based on the observed formant values.
(TIF)

Figure S2 **Spectrograms and power spectra to show examples of rumbles from a 43 year old female African elephant (Jumbo) recorded at the Vienna Zoo in 2003 (using the same equipment as described in Figure S1).** Figures A and B also show a rumble recorded during spatial separation from the group, again with the formant structure of a typical nasal rumble. Figures C and D show a rumble recorded during the bonding situation when the group was reunited, again resembling an orally emitted rumble based on the formant values. Jumbo died in 2004 and her oral vocal tract was measuring at 93 cm (Weissengruber, personal communication). The formants 1 and 2 of the oral vocal tract would thus be (using equation 3) 92 Hz and 277 Hz, which corresponds very well with the formant location observed in Figures C and D.
(TIF)
