# Supplementary figures and images for: Visualizing Sound Emission of Elephant Vocalizations: Evidence for Two Rumble Production Types
Source: PLoS One. 2012 Nov 14;7(11):e48907. doi: 10.1371/journal.pone.0048907 (PMC3498347; doi:10.1371/journal.pone.0048907)

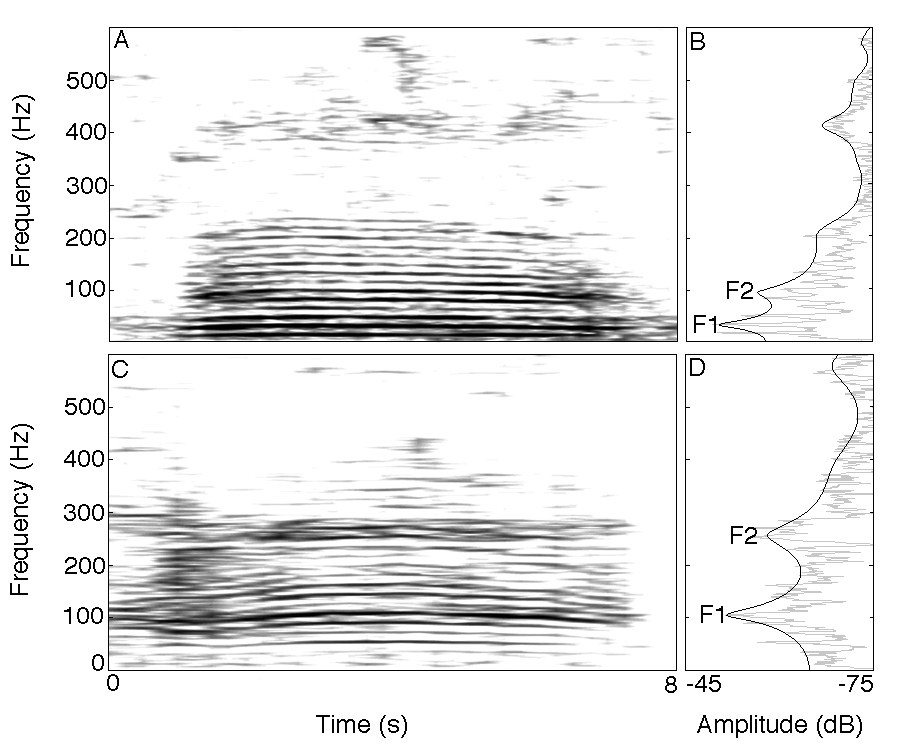

Supplement: Figure S1 — Spectrograms and power spectra presenting two examples of rumbling vocalizations from a 29 year old female African elephant (Drumbo) recorded at the Vienna Zoo in 2003. Recordings were captured with a condenser microphone AKG 480 B CK 62 and a DA-P1 DAT recorder. Figures A and B show a rumble recorded during spatial separation from a part of the group, and display the formant structure of a typical nasal rumble. Figures C and D show a rumble recorded during a bonding situation when the group was reunited, and resemble an orally emitted rumble based on the observed formant values. (TIF) [file pone.0048907.s005.tif]

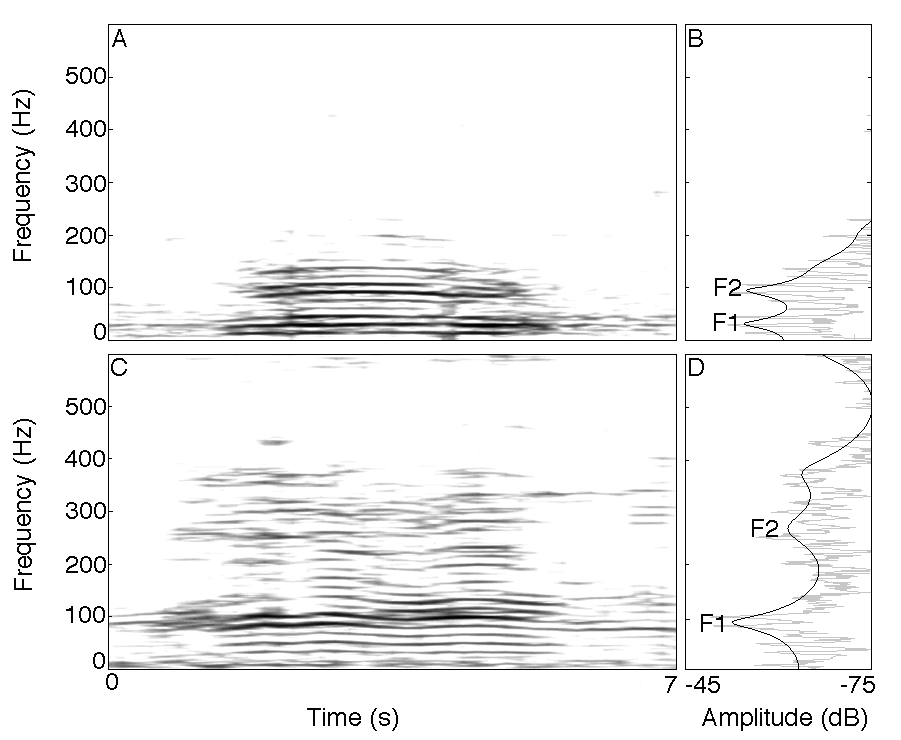

Supplement: Figure S2 — Spectrograms and power spectra to show examples of rumbles from a 43 year old female African elephant (Jumbo) recorded at the Vienna Zoo in 2003 (using the same equipment as described in Figure S1). Figures A and B also show a rumble recorded during spatial separation from the group, again with the formant structure of a typical nasal rumble. Figures C and D show a rumble recorded during the bonding situation when the group was reunited, again resembling an orally emitted rumble based on the formant values. Jumbo died in 2004 and her oral vocal tract was measuring at 93 cm (Weissengruber, personal communication). The formants 1 and 2 of the oral vocal tract would thus be (using equation 3) 92 Hz and 277 Hz, which corresponds very well with the formant location observed in Figures C and D. (TIF) [file pone.0048907.s006.tif]
